# Irisin and endocrine-metabolic indicators as predictors of IVF-ET pregnancy outcomes in Chinese women with PCOS

**DOI:** 10.3389/fendo.2026.1894019

**Published:** 2026-08-14

**Authors:** Huixia Wang, Chunlin Su, Wei Jin, Weiwei Lu, Tingting Bi, Qilin Cheng, Dongmei Ji

**Affiliations:** 1 Department of Obstetrics and Gynecology, Fifth People’s Hospital of Huai’an, Jiangsu, Huai’an, China; 2 Department of Gynecological Endocrinology and Reproductive Medicine, Obstetrics and Gynecology Hospital Affiliated to Fudan University, Shanghai, China

**Keywords:** *in vitro* fertilization-embryo transfer, insulin resistance, logistic regression analysis, polycystic ovary syndrome, pregnancy outcome, reproductive endocrinology

## Abstract

**Objective:**

To evaluate the association of serum irisin, together with reproductive endocrine and metabolic indicators, with clinical pregnancy outcomes following *in vitro* fertilization and embryo transfer (IVF-ET) in women with polycystic ovary syndrome (PCOS).

**Methods:**

This single-center retrospective cohort study included 92 infertile PCOS patients who underwent their first IVF-ET treatment at our hospital from February 2022 to February 2024. Available baseline data, IVF-related treatment parameters, reproductive endocrine and metabolic indicators were retrospectively collected from the medical records, and pregnancy outcomes were followed up. Univariate analysis and multivariate logistic regression models were used to evaluate independent factors affecting pregnancy outcomes. Receiver operating characteristic (ROC) curve analysis was conducted to assess the predictive value of related indicators.

**Results:**

The pregnancy success group (n=44) was younger, required a lower total dose of gonadotropins (Gn), and retrieved more oocytes (P<0.05). Regarding reproductive endocrine parameters, the success group had higher levels of follicle-stimulating hormone (FSH), progesterone (P), and sex hormone-binding globulin (SHBG), and a lower luteinizing hormone (LH)/FSH ratio (P<0.05). Metabolically, the success group showed significantly lower fasting blood glucose (FBG), fasting insulin (FINS), homeostasis model assessment of insulin resistance (HOMA-IR), triglycerides (TG), total cholesterol (TC), and low-density lipoprotein cholesterol (LDL-C), but higher high-density lipoprotein cholesterol (HDL-C) levels compared with the failure group (P<0.001). Multivariate logistic regression indicated that LH/FSH ratio (OR = 0.616), free testosterone index (FTI, OR = 0.363), irisin (OR = 0.401), and Gn dosage (OR = 0.445) were independent factors associated with lower odds of clinical pregnancy success (P<0.05). ROC curve analysis suggested that LH/FSH ratio, free testosterone index (FTI), irisin, and Gn dosage had moderate predictive value for pregnancy outcomes.

**Conclusion:**

The reproductive endocrine and metabolic characteristics of PCOS patients partially influence pregnancy outcomes following IVF-ET. Among the available endocrine, metabolic, and treatment-related indicators, LH/FSH ratio, FTI, irisin, and Gn dosage may serve as reference indicators for predicting IVF-ET pregnancy outcomes, suggesting that a more favorable endocrine and metabolic profile may be related to improved clinical pregnancy probability.

## 1 Introduction

Polycystic Ovary Syndrome (PCOS) is one of the most common endocrine disorders affecting women of reproductive age, with a prevalence of approximately 5% to 15%, and accounting for 30% to 40% of infertility cases among women (1, 2). Its main features include oligo-ovulation, hyperandrogenemia, and polycystic ovarian morphology, with clinical manifestations varying across different phenotypes, often accompanied by obesity, oligomenorrhea or amenorrhea, hirsutism, and acne, which severely impact patients’ fertility and quality of life. The etiology of PCOS is complex and not completely understood, primarily involving dysfunction of the hypothalamic-pituitary-ovarian axis, insulin resistance, genetic factors, and environmental influences (3, 4).

Besides causing ovulatory infertility, PCOS patients face higher risks of adverse pregnancy outcomes even after conception, such as spontaneous miscarriage, gestational diabetes mellitus, preterm birth, and low birth weight (5). These unfavorable pregnancy outcomes may be closely related to decreased oocyte quality, impaired embryonic developmental potential, and reduced endometrial receptivity. Studies have shown that elevated luteinizing hormone (LH) levels and a hyperandrogenic environment can cause abnormal expression of estrogen and progesterone receptors in the endometrium, thereby affecting embryo implantation and pregnancy maintenance (6, 7).

With continuous advances in assisted reproductive technology, *in vitro* fertilization and embryo transfer (IVF-ET) has become an important treatment modality for infertility associated with PCOS. Although IVF-ET has achieved positive results in ovulation induction and fertilization, PCOS patients still face several challenges during IVF cycles, such as low oocyte maturation rate, poor embryo quality, inadequate endometrial development, and ovarian hyperstimulation syndrome (OHSS) (8). Moreover, pregnancy outcomes in PCOS patients are influenced by multiple endocrine and metabolic factors, with inconsistent findings across studies. For example, some research suggests that high body mass index (BMI) does not significantly affect IVF outcomes in Chinese PCOS patients (9), while other studies report that lean-type PCOS patients have higher clinical pregnancy success rates (10).

Furthermore, the prevalence of metabolic syndrome in PCOS patients approaches 30%, characterized by insulin resistance, impaired glucose tolerance, and dyslipidemia. These metabolic abnormalities may affect oocyte and embryo quality and increase the risks of miscarriage and preterm birth by regulating apoptosis and the expression of genes related to follicular development (11, 12). Irisin, a novel exercise-induced myokine, has emerged as an important regulator of energy metabolism and insulin sensitivity and may therefore be closely related to the metabolic features of PCOS (13). Recent evidence further suggests that irisin participates in reproductive regulation and may serve as a potential biomarker linking metabolic dysfunction with reproductive outcomes in PCOS (14). In addition, androgen-related indicators such as the free testosterone index (FTI), together with other endocrine and metabolic markers, have attracted increasing attention in the evaluation of ovarian function and pregnancy outcomes; however, their clinical applications in IVF-ET outcome prediction remain insufficiently validated (15, 16). Therefore, further evaluation of irisin together with conventional endocrine and metabolic indicators may help clarify its practical predictive value in PCOS patients undergoing IVF-ET.

Therefore, it is clinically important to evaluate candidate biomarkers in the context of routinely available endocrine, metabolic, and treatment-related variables in PCOS patients undergoing IVF-ET. Rather than re-establishing the general biological role of irisin in insulin sensitivity or fertility, the present study focused on whether serum irisin is associated with IVF-ET pregnancy outcomes when analyzed alongside conventional indicators such as BMI, LH/FSH ratio, FTI, metabolic parameters, Gn dosage, and oocyte retrieval outcomes. Based on this rationale, we retrospectively analyzed available clinical data from infertile PCOS patients undergoing their first IVF-ET cycle and assessed the potential predictive value of irisin and related endocrine-metabolic indicators for clinical pregnancy outcomes.

## 2 Subjects and methods

### 2.1 Study subjects

This retrospective single-center cohort study included 92 infertile patients with polycystic ovary syndrome (PCOS) who underwent their first *in vitro* fertilization and embryo transfer (IVF-ET) cycle at the Reproductive Medicine Center of our hospital between February 2022 and February 2024. PCOS was diagnosed according to the current domestic “Guidelines or Expert Consensus on Diagnosis and Treatment of Polycystic Ovary Syndrome (PCOS)” (15). The inclusion criteria were as follows: (1) age between 25 and 36 years; (2) infertility duration of ≥12 months; and (3) normal semen parameters of the spouse according to WHO standards, with severe oligozoospermia, asthenozoospermia, or other male factors excluded. The exclusion criteria were as follows: (1) concomitant diabetes, cardiovascular disease, or organic endometrial lesions; (2) other endocrine disorders causing hyperandrogenemia, such as adrenal hyperplasia or Cushing’s syndrome; (3) history of hypertensive disorders during pregnancy or thyroid dysfunction; and (4) cancellation of the IVF-ET cycle due to failure to retrieve oocytes, fertilization failure, or absence of transferable embryos.

Because this was a retrospective cohort study, no formal *a priori* sample size calculation was performed. The sample size was determined by the number of eligible PCOS patients who underwent their first IVF-ET cycle during the predefined study period and met the inclusion and exclusion criteria. Finally, 92 patients were included, comprising 44 patients with clinical pregnancy success and 48 with pregnancy failure. For the multivariate logistic regression analysis, the final model retained four predictors, including LH/FSH ratio, FTI, irisin, and Gn dose. The 44 clinical pregnancy events corresponded to approximately 11 events per variable, which was consistent with the commonly used criterion of at least 10 events per variable for exploratory logistic regression analysis. Therefore, the cohort size was considered acceptable for preliminary multivariable analysis, although validation in larger prospective cohorts remains necessary.

This retrospective study was reviewed and approved by the Ethics Committee of the Fifth People’s Hospital of Huai’an City (Approval No.: 2021-9872). All patients had provided written informed consent for IVF-ET treatment before treatment as part of routine clinical practice. For the present retrospective analysis, only de-identified clinical data were extracted from existing medical records, and no additional intervention, examination, follow-up procedure, or risk was imposed on patients. Therefore, the requirement for additional study-specific informed consent was waived by the ethics committee.

### 2.2 IVF-ET treatment protocol

All enrolled patients underwent a standardized ovulation induction protocol: a long luteal phase gonadotropin-releasing hormone agonist (GnRH-a) protocol following oral contraceptive pretreatment. Specifically, GnRH-a was administered by intramuscular injection in the mid-luteal phase of the menstrual cycle for pituitary downregulation. After downregulation criteria were met (usually 14–18 days post-administration), controlled ovarian stimulation (COS) with gonadotropin (Gn) injections was initiated on day 3 of the treatment cycle. When ultrasound monitoring showed any of the following dominant follicle criteria: one follicle ≥18 mm, or two follicles ≥17 mm, or three follicles ≥16 mm in diameter, human chorionic gonadotropin (hCG) 5,000–10,000 IU was injected intramuscularly that evening to trigger final oocyte maturation. Serum irisin levels were measured using a commercially available enzyme-linked immunosorbent assay (ELISA) kit (BioVendor, Brno, Czech Republic, Catalog No.: RAG018R) according to the manufacturer’s instructions. The same fasting serum samples collected on day 3 of the menstrual cycle were used for irisin measurement. All samples were processed in duplicate. According to the manufacturer’s specifications, the assay has a detection range of 0.001–5μg/mL, with a limit of detection of 1 ng/mL. The intra-assay and inter-assay coefficients of variation were <6.9% and <9.1%, respectively, indicating good assay precision.

Oocyte retrieval was performed 36 ± 1 hours after hCG injection under transvaginal ultrasound guidance. Retrieved oocytes were cultured *in vitro* for 4–6 hours before conventional IVF fertilization. Embryos were cultured until 72 hours post-retrieval (cleavage stage). In accordance with the routine protocol of our center, two transferable embryos with the best available morphological appearance were selected for intrauterine transfer, and cycles without transferable embryos were excluded. Therefore, the number of transferred embryos was relatively standardized in this cohort; however, detailed embryo-grade strata and implantation scores were not available in a complete structured format for multivariable adjustment. Luteal support was provided with daily intramuscular progesterone injections starting from the day of oocyte retrieval until 14 days after embryo transfer.

Biochemical pregnancy was determined by serum or urine hCG testing 14 days post-transfer. Clinical pregnancy was confirmed by transvaginal ultrasound detecting an intrauterine gestational sac 28–30 days after transfer.

### 2.3 Data collection and observational indicators

The following data were retrospectively collected from the electronic medical records. All extracted data were de-identified before statistical analysis.

Baseline data: age, duration of infertility, body mass index (BMI).

Treatment-related parameters: total Gn dose and total number of oocytes retrieved. Because this retrospective study used routinely archived clinical records, detailed endometrial thickness on the trigger day or embryo-transfer day, embryo quality grades, and embryo implantation scores were not consistently available in analyzable form across all patients.

Serum reproductive endocrine and metabolic indicators:

All patients had fasting venous blood (5 mL) collected from the antecubital vein on day 3 of menstruation (or day 3 after progesterone withdrawal in amenorrheic patients) following at least 8 hours of fasting in the morning. Samples were allowed to clot at room temperature for 30 minutes, then centrifuged at 4 °C, 4500 rpm for 5 minutes using a low-temperature high-speed centrifuge (Thermo Scientific). Serum levels of follicle-stimulating hormone (FSH), luteinizing hormone (LH), estradiol (E2), prolactin (PRL), sex hormone-binding globulin (SHBG), progesterone (P), testosterone (T), triglycerides (TG), total cholesterol (TC), high-density lipoprotein cholesterol (HDL-C), and low-density lipoprotein cholesterol (LDL-C) were measured using a Siemens ADVIA 2400 automatic biochemical analyzer. The LH/FSH ratio was calculated. The Free Testosterone Index (FTI) was calculated as follows: FTI = (Testosterone [nmol/L]/SHBG [nmol/L]) × 100. FTI was treated as a dimensionless index in subsequent analyses.

Fasting insulin (FINS) was measured by electrochemiluminescence immunoassay on a Roche Cobas e 411 analyzer using matched reagent kits (Insulin Detection Kit). Fasting blood glucose (FBG) was determined using the glucose hexokinase method. Insulin resistance was estimated by the homeostasis model assessment of insulin resistance (HOMA-IR) calculated as:


HOMA−IR = [FBG (mmol/L) × FINS (μIU/mL)]/22.5.

IVF-ET pregnancy outcomes: Pregnancy outcomes were followed by regular outpatient visits, with main endpoints including clinical pregnancy, miscarriage, ectopic pregnancy, and live birth. Clinical pregnancy was defined as the presence of an intrauterine gestational sac confirmed by transvaginal ultrasound within 30–40 days after embryo transfer. Miscarriage was defined as pregnancy loss before 28 weeks gestation or fetal weight below 1000 grams leading to pregnancy termination. Live birth was defined as delivery of a live newborn at ≥28 weeks gestation.

### 2.4 Statistical analysis

Data processing and analysis were performed using SPSS software version 19.0. Normally distributed continuous variables were expressed as mean ± standard deviation (mean ± SD) and compared between groups using two-tailed independent samples t-test. Non-normally distributed data were expressed as median (interquartile range) [M (P25, P75)] and compared using the Mann-Whitney U test. Categorical variables were described as number (percentage) [n (%)] and compared using the chi-square test or Fisher’s exact test when expected counts were <5.

To explore whether irisin and routinely available reproductive endocrine, metabolic, and treatment-related variables were independently associated with IVF-ET pregnancy outcomes, a multivariate binary logistic regression model was constructed. Clinical pregnancy outcome was coded as success=1 and failure=0; therefore, odds ratios were interpreted in relation to the odds of clinical pregnancy success. Univariate analysis was first conducted to screen baseline variables potentially associated with pregnancy outcome (P<0.10) or of known clinical significance (e.g., FSH, LH, LH/FSH, T, SHBG, HOMA-IR, blood lipids) for inclusion in the initial model. Among variables consistently available in the retrospective dataset, clinically important covariates including age, BMI, total Gn dose, and total number of oocytes retrieved were entered into the model to adjust for potential confounding effects. BMI was entered as a continuous covariate. Additional BMI-stratified analyses (<24 *vs*. ≥24 kg/m²) and nonlinear analyses using restricted cubic splines were not performed because of the limited sample size, narrow BMI distribution, and insufficient number of patients with obesity. Therefore, the multivariate model was interpreted as an exploratory model based on available clinical, endocrine, metabolic, and treatment-related variables. To reduce the risk of model overfitting, the number of variables retained in the final model was restricted according to the available number of clinical pregnancy events. Therefore, the multivariate model was interpreted as an exploratory model based on available clinical, endocrine, metabolic, and treatment-related variables. Variable selection was performed using the forward likelihood ratio method (Forward: LR). The model fit was assessed using the Hosmer-Lemeshow goodness-of-fit test, with a non-significant P-value (P>0.05) indicating adequate fit. The Akaike Information Criterion (AIC) was calculated to evaluate model quality, with lower values indicating better model fit. Results were presented as adjusted odds ratios (OR) with 95% confidence intervals (95% CI).

Furthermore, receiver operating characteristic (ROC) curve analysis was performed to evaluate the predictive performance of key indicators identified in the final model, including LH/FSH ratio, FTI, irisin, and Gn dose. The area under the curve (AUC), optimal cutoff value, sensitivity, and specificity were calculated. All tests were two-tailed, with P<0.05 considered statistically significant.

## 3 Results

### 3.1 Comparison of basic characteristics and treatment parameters

Comparison between the pregnancy success group (n=44) and pregnancy failure group (n=48) revealed significant differences in age, gonadotropin (Gn) dosage, and total number of oocytes retrieved. The pregnancy success group was younger than the failure group (29.12 ± 3.05 *vs*. 30.23 ± 3.18 years, t=2.295, P = 0.023), received a lower total Gn dose (2265.72 ± 195.33 *vs*. 2388.22 ± 215.65 U, t=2.388, P = 0.018), but had a higher total number of oocytes retrieved (10.55 ± 2.08 *vs*. 9.12 ± 1.32, t=4.455, P<0.001). No significant differences were found in body mass index (BMI) or duration of infertility between the two groups (P>0.05) (**Table 1**). The mean BMI values in both groups were close to 24 kg/m², indicating a relatively narrow BMI distribution in this cohort.

**Table 1:** Comparison of basic characteristics and treatment parameters between the pregnancy success and failure groups.

Parameter	Pregnancy success group (n=44)	Pregnancy failure group (n=48)	t	P
Age (years)	29.12 ± 3.05	30.23 ± 3.18	2.295	0.023
BMI (kg/m²)	23.78 ± 1.76	24.02 ± 1.55	0.252	0.819
Duration of infertility (years)	3.22 ± 0.68	2.98 ± 0.55	1.021	0.313
Total Gn dose (U)	2265.72 ± 195.33	2388.22 ± 215.65	2.388	0.018
Total oocytes retrieved (count)	10.55 ± 2.08	9.12 ± 1.32	4.455	<0.001

Data are presented as mean ± standard deviation. The pregnancy success group included patients with clinical pregnancy confirmed by transvaginal ultrasound after embryo transfer, whereas the pregnancy failure group included patients without clinical pregnancy. Between-group comparisons were performed using the independent-samples t-test. BMI, body mass index; Gn, gonadotropin; IVF-ET, *in vitro* fertilization and embryo transfer. A two-sided P<0.05 was considered statistically significant.

### 3.2 Comparison of reproductive endocrine and metabolic indicators

Regarding reproductive endocrine parameters, the pregnancy success group had significantly higher FSH levels compared to the failure group (6.18 ± 0.92 *vs*. 5.85 ± 0.85 IU/L, P = 0.003), while the LH/FSH ratio was markedly lower (1.22 ± 0.12 *vs*. 1.45 ± 0.10, P<0.001). Additionally, progesterone (P) levels were higher in the success group (2.15 ± 0.45 *vs*. 1.88 ± 0.38 nmol/L, P = 0.002), and sex hormone-binding globulin (SHBG) levels were significantly elevated (41.15 ± 7.22 *vs*. 32.42 ± 6.05 nmol/L, P<0.001). Notably, prolactin (PRL) levels were significantly lower in the pregnancy success group compared to the failure group (15.2 ± 3.5 *vs*. 22.8 ± 4.1 μg/L, P<0.001). There were no significant differences in estradiol (E_2_), luteinizing hormone (LH), and testosterone (T) levels between the groups (P>0.05).

Metabolically, the pregnancy success group exhibited significantly lower levels of fasting blood glucose (FBG), fasting insulin (FINS), insulin resistance index (HOMA-IR), triglycerides (TG), total cholesterol (TC), and low-density lipoprotein cholesterol (LDL-C) compared to the failure group (all P<0.001), while high-density lipoprotein cholesterol (HDL-C) was higher (1.35 ± 0.22 *vs*. 1.08 ± 0.18 mmol/L, P<0.001) (**Table 2**).

**Table 2:** Comparison of reproductive endocrine and metabolic indicators between the pregnancy success and failure groups.

Parameter	Pregnancy success group (n=44)	Pregnancy failure group (n=48)	t	P
FSH (IU/L)	6.18 ± 0.92	5.85 ± 0.85	0.698	0.003
LH (IU/L)	7.72 ± 1.18	8.22 ± 1.48	1.055	0.302
LH/FSH	1.22 ± 0.12	1.45 ± 0.10	4.812	<0.001
E_2_ (pmol/L)	206.55 ± 40.88	195.22 ± 26.95	1.705	0.089
PRL (μg/L)	15.2 ± 3.5	22.8 ± 4.1	8.774	<0.001
T (nmol/L)	1.10 ± 0.22	1.17 ± 0.18	1.212	0.234
P (nmol/L)	2.15 ± 0.45	1.88 ± 0.38	3.112	0.002
SHBG (nmol/L)	41.15 ± 7.22	32.42 ± 6.05	6.333	<0.001
FTI	2.67 ± 0.55	3.61 ± 0.72	7.215	<0.001
FBG (mmol/L)	4.85 ± 0.35	5.25 ± 0.42	5.122	<0.001
FINS (μIU/mL)	10.2 ± 2.5	18.6 ± 3.8	12.76	<0.001
HOMA-IR	1.8 ± 0.6	3.2 ± 0.9	10.417	<0.001
TG (mmol/L)	1.25 ± 0.28	1.88 ± 0.35	9.226	<0.001
TC (mmol/L)	4.25 ± 0.52	4.98 ± 0.61	6.43	<0.001
HDL-C (mmol/L)	1.35 ± 0.22	1.08 ± 0.18	6.912	<0.001
LDL-C (mmol/L)	2.35 ± 0.38	3.05 ± 0.42	8.32	<0.001

Data are presented as mean ± standard deviation. The pregnancy success group included patients with clinical pregnancy confirmed by transvaginal ultrasound after embryo transfer, whereas the pregnancy failure group included patients without clinical pregnancy. Between-group comparisons were performed using the independent-samples t-test. FSH, follicle-stimulating hormone; LH, luteinizing hormone; E_2_, estradiol; PRL, prolactin; T, testosterone; P, progesterone; SHBG, sex hormone-binding globulin; FTI, free testosterone index; FBG, fasting blood glucose; FINS, fasting insulin; HOMA-IR, homeostasis model assessment of insulin resistance; TG, triglycerides; TC, total cholesterol; HDL-C, high-density lipoprotein cholesterol; LDL-C, low-density lipoprotein cholesterol. FTI was calculated as testosterone (nmol/L)/SHBG (nmol/L) × 100 and is dimensionless. HOMA-IR was calculated as FBG (mmol/L) × FINS (μIU/mL)/22.5. A two-sided P<0.05 was considered statistically significant.

### 3.3 Multivariate logistic regression analysis of factors affecting pregnancy outcomes

**Figure 1:**
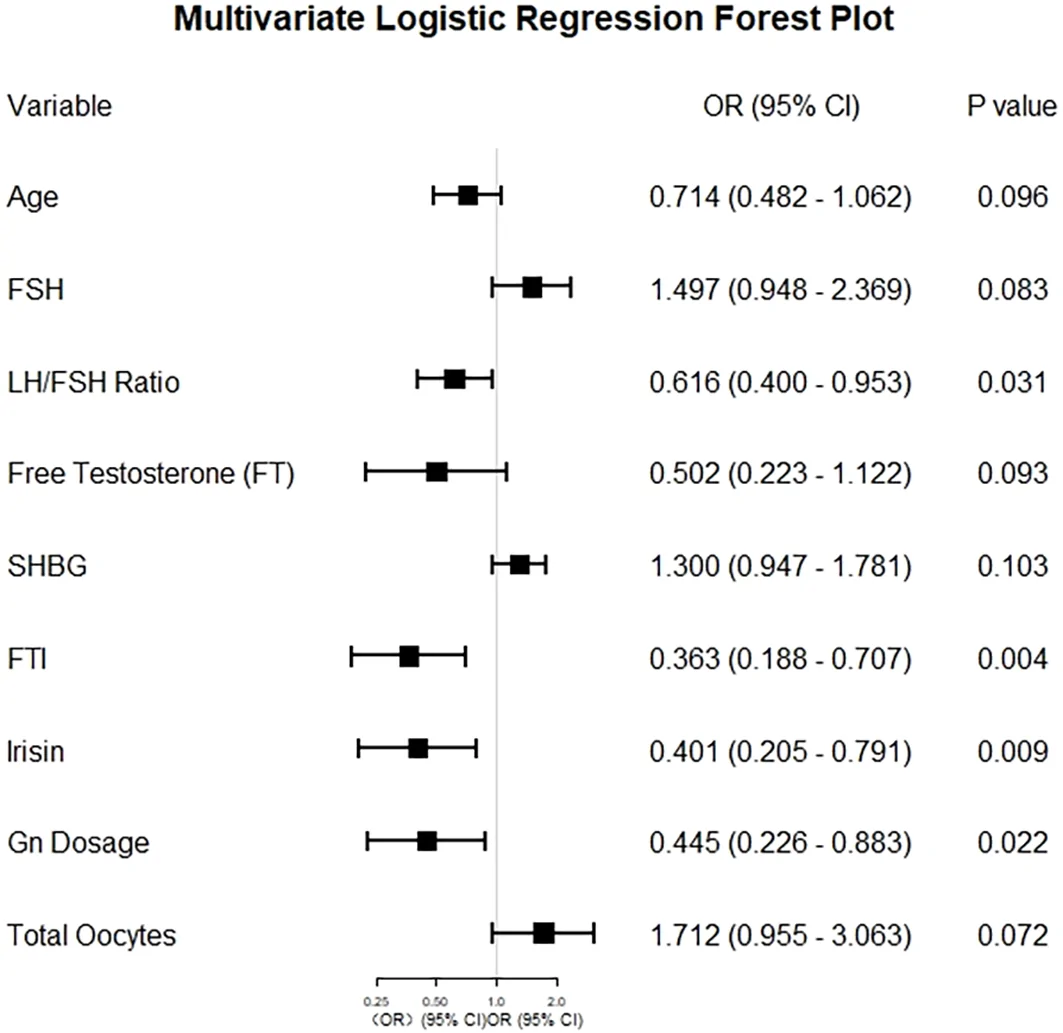
Forest plot of multivariate logistic regression analysis for factors affecting pregnancy outcomes. The model was adjusted for age, BMI, total Gn dose, and total number of oocytes retrieved. Odds ratios (OR) and 95% confidence intervals are shown. The final model demonstrated good fit, as indicated by a non-significant Hosmer-Lemeshow test (P = 0.612) and an Akaike Information Criterion (AIC) of 98.45.

Multivariate logistic regression analysis was performed to identify factors influencing pregnancy outcomes, with results presented in the forest plot (**Figure 1**). The final model showed good fit with a Hosmer-Lemeshow test χ² = 6.32 (P = 0.612) and Akaike Information Criterion (AIC) = 98.45. The analysis showed that the LH/FSH ratio (OR = 0.616, 95% CI: 0.400–0.953, P = 0.031), free testosterone index (FTI, OR = 0.363, 95% CI: 0.188–0.707, P = 0.004), irisin (OR = 0.401, 95% CI: 0.205–0.791, P = 0.009), and gonadotropin (Gn) dose (OR = 0.445, 95% CI: 0.226–0.883, P = 0.022) were independently associated with clinical pregnancy outcomes. As the dependent variable was clinical pregnancy success, these OR values below 1 indicate that higher levels of these indicators were associated with lower odds of clinical pregnancy success.

Other variables considered in the model, including age, follicle-stimulating hormone (FSH), testosterone (T), sex hormone-binding globulin (SHBG), and total oocytes retrieved, did not reach statistical significance (P>0.05). We acknowledge that age and Gn dose differences, while observed in our cohort, are known to influence pregnancy outcomes in both PCOS and non-PCOS populations. The modest AUC values obtained in our ROC analysis (ranging from 0.663 to 0.764) may reflect the complex multifactorial nature of IVF-ET outcomes in PCOS patients, as indicated by the AIC value of 98.45, which suggests adequate but not perfect model fit given the sample size. To address the potential interplay between LH/FSH ratio and FTI, we performed correlation analysis which revealed a weak but statistically significant positive correlation between LH/FSH ratio and FTI (r = 0.248, P = 0.018), suggesting these factors may operate through partially overlapping but distinct pathways in influencing pregnancy outcomes in PCOS patients. These findings indicate that, within the available retrospective variables and the exploratory sample size of this cohort, LH/FSH ratio, FTI, irisin, and Gn dose may serve as predictive indicators of IVF-ET pregnancy outcomes in PCOS patients, providing a basis for further evaluation of endocrine and metabolic risk profiles before treatment.

### 3.4 ROC curve analysis of factors influencing pregnancy outcomes

**Figure 2:**
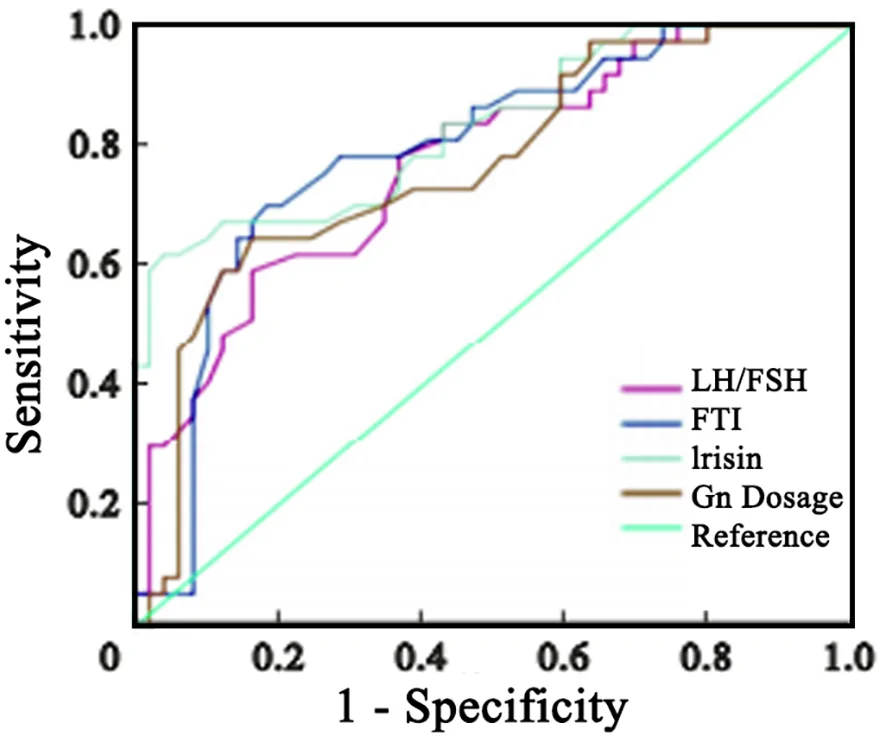
Receiver operating characteristic (ROC) curve analysis of LH/FSH ratio, FTI, irisin, and Gn dose for predicting IVF-ET pregnancy outcomes.

Receiver operating characteristic (ROC) curves were generated to assess the predictive value of key factors for IVF-ET pregnancy outcomes in PCOS patients (**Figure 2**). The optimal cut-off values for predicting pregnancy outcomes were 1.35 for LH/FSH ratio, 3.22 for FTI, 210.28 μg/L for irisin, and 2325 U for Gn dose.

The area under the curve (AUC) and Youden index were calculated to evaluate the predictive performance of these indicators. LH/FSH ratio showed an AUC of 0.732 (95% CI: 0.645–0.819) with a Youden index of 0.428; at a cut-off value of 1.35, the sensitivity and specificity were 71.5% and 71.3%, respectively, indicating moderate predictive value. FTI demonstrated an AUC of 0.685 (95% CI: 0.592–0.778) and a Youden index of 0.372; at a cut-off value of 3.22, the sensitivity was 65.2% and specificity was 71.8%, suggesting auxiliary predictive value. Irisin exhibited the best predictive performance among the single indicators, with an AUC of 0.764 (95% CI: 0.679–0.849) and a Youden index of 0.483; at a cut-off value of 210.28 μg/L, the sensitivity reached 73.8% and specificity reached 74.5%. The gonadotropin (Gn) dose had an AUC of 0.663 (95% CI: 0.570–0.756) and a Youden index of 0.341; at a cut-off value of 2325 U, the sensitivity and specificity were 62.7% and 71.4%, respectively.

Overall, LH/FSH ratio, FTI, irisin, and Gn dose showed moderate predictive value for IVF-ET pregnancy outcomes in PCOS patients. These findings suggest that a predictive model integrating endocrine, metabolic, and treatment-related indicators may be more informative than any single marker alone.

## 4 Discussion

This retrospective study analyzed available clinical data from 92 infertile patients with PCOS undergoing their first IVF-ET treatment, with particular attention to irisin within a broader endocrine-metabolic framework. The results showed that several baseline endocrine, metabolic, and treatment-related variables were associated with clinical pregnancy outcomes, including age, Gn dose, number of oocytes retrieved, LH/FSH ratio, FTI, insulin resistance-related indicators, lipid metabolism indicators, and irisin. In the multivariate model, irisin remained associated with clinical pregnancy outcome together with LH/FSH ratio, FTI, and Gn dose, suggesting that irisin may provide clinical predictive information when interpreted alongside conventional endocrine and metabolic parameters rather than as an isolated marker.

Specifically, the LH/FSH ratio was significantly lower in the pregnancy success group compared with the failure group (1.22 *vs*. 1.45, P < 0.001) and remained significant in multivariate regression (OR = 0.616, P = 0.031), suggesting that an elevated LH/FSH ratio may be unfavorably associated with clinical pregnancy success. Elevated LH/FSH ratio is a common endocrine characteristic in PCOS patients, reflecting dysregulation of the hypothalamic-pituitary-ovarian axis. This imbalance can lead to arrested follicular development and decreased oocyte quality, thereby affecting embryo implantation and pregnancy rates (16, 17). Previous studies have suggested that an LH/FSH ratio > 2 serves as a sensitive indicator for increased risk of IVF-ET failure, highlighting the clinical significance of dynamic monitoring and timely intervention (8).

Additionally, the free testosterone index (FTI) was identified as an independent factor unfavorably associated with clinical pregnancy success (OR = 0.363, P = 0.004), supporting the important role of androgen bioactivity in reproductive outcomes. High FTI reflects increased androgen bioactivity, and hyperandrogenism not only disrupts the follicular microenvironment but may also impair granulosa cell function, resulting in follicular atresia and embryonic development disorders (18, 19). Related research has confirmed that excess androgens exacerbate insulin resistance by inhibiting insulin signaling pathways, compounding negative endocrine and metabolic effects (3).

The BMI findings in this study should be interpreted cautiously. Although BMI did not differ significantly between the pregnancy success and failure groups, the mean BMI values in both groups were close to 24 kg/m², and the cohort did not include a sufficient number of patients with obesity (BMI ≥28 kg/m²) for meaningful obesity subgroup analysis. Therefore, the present data cannot determine whether obesity itself affects IVF-ET outcomes in PCOS patients. Rather, within this relatively narrow BMI range, endocrine and metabolic indicators, including insulin resistance and dyslipidemia, showed clearer associations with clinical pregnancy outcomes than BMI. This suggests that metabolic status may provide additional information beyond BMI in this cohort, but it should not be interpreted as evidence that obesity is unimportant. Previous studies have reported inconsistent findings regarding the effect of BMI or obesity on IVF outcomes in PCOS patients (9, 20), further supporting the need for studies with broader BMI distributions and larger sample sizes.

The rationale for focusing on irisin lies not in re-confirming its established involvement in insulin sensitivity or reproductive regulation, but in assessing its potential clinical value in IVF-ET outcome prediction among PCOS patients. In the present study, irisin was retained in the final model (OR = 0.401, P = 0.009), suggesting that it may be a potential biomarker associated with IVF-ET pregnancy outcomes when evaluated together with endocrine, metabolic, and treatment-related variables. Irisin is a myokine involved in energy metabolism, insulin sensitivity, and inflammatory regulation (21). However, the direction of association observed in the present study suggests that higher circulating irisin levels were not necessarily linked to better pregnancy outcomes. One possible explanation is that increased irisin may reflect a compensatory response to underlying metabolic stress, insulin resistance, or endocrine dysregulation in PCOS rather than a direct beneficial effect. Previous studies have also indicated that the relationship between irisin, metabolic status, and reproductive function is complex and may vary according to patient phenotype and metabolic background (13, 22, 23). Therefore, irisin should be interpreted as a predictive biomarker rather than as a confirmed protective factor in this retrospective cohort. Further prospective studies are needed to clarify its causal role and biological mechanisms in PCOS patients undergoing IVF-ET.

Meanwhile, Gn dose was also an important treatment-related variable associated with pregnancy outcomes. Even after controlling for potential confounders such as age and oocyte number, higher total Gn dosage remained associated with lower odds of clinical pregnancy success (OR = 0.445, P = 0.022). The pregnancy success group used lower Gn doses, suggesting that better ovarian responsiveness or more appropriate stimulation protocols may help obtain higher quality oocytes and embryos. This conclusion aligns with previous studies, which have indicated that excessive Gn stimulation can cause ovarian hyper-response and interfere with endometrial receptivity, thus reducing pregnancy rates (24). Moreover, Gn requirements are often influenced by patients’ insulin resistance status, with higher HOMA-IR values correlating to increased Gn demand but poorer oocyte quality. Therefore, individualized Gn dosing tailored to patients’ metabolic profiles may improve assisted reproduction success rates (25). It should also be noted that IVF-ET outcomes are determined not only by baseline endocrine and metabolic status but also by endometrial receptivity and embryological factors. In routine clinical practice, endometrial thickness around trigger or embryo transfer, the number and quality of transferred embryos, and embryo implantation scores may substantially influence clinical pregnancy. In the present cohort, the number of transferred embryos was relatively standardized by the routine transfer protocol, whereas detailed endometrial thickness, embryo-grade categories, and implantation scores were not consistently available in structured retrospective records. Therefore, these variables could not be incorporated into the multivariate model. Their absence may have introduced residual confounding, and the associations observed for LH/FSH ratio, FTI, irisin, and Gn dose should be interpreted as findings based on available retrospective variables rather than as a complete prediction model for IVF-ET outcomes.

Insulin resistance also showed a clear indication in this study. The pregnancy success group demonstrated significantly better insulin sensitivity, as reflected by lower HOMA-IR, fasting insulin (FINS), and fasting blood glucose (FBG) levels compared to the failure group. This result is consistent with extensive literature showing that insulin resistance interferes with ovulation and embryo implantation through multiple mechanisms (1). On one hand, it promotes androgen synthesis in ovarian stromal cells, disrupting gonadotropin secretion; on the other hand, it may affect uterine blood flow and cellular growth, reducing endometrial receptivity (26). Meanwhile, SHBG levels were significantly elevated in the pregnancy success group, further supporting its negative correlation with insulin status. As a protein markedly suppressed by insulin, SHBG concentration serves as an indirect marker of insulin resistance (27).

Another key metabolic finding was the difference in blood lipid levels. The pregnancy success group had significantly lower triglycerides (TG), total cholesterol (TC), and low-density lipoprotein cholesterol (LDL-C), and higher high-density lipoprotein cholesterol (HDL-C), indicating that a favorable lipid metabolism profile may support embryo implantation and development (11). PCOS patients often suffer from metabolic syndrome, where dyslipidemia affects systemic metabolic homeostasis and negatively impacts oocyte and embryo quality through oxidative stress and local inflammation (28, 29). Thus, interventions aimed at improving lipid metabolism may offer new approaches to enhance IVF outcomes.

An intriguing finding of our study was the significantly lower serum prolactin (PRL) level in the pregnancy success group. While PRL is primarily known for its role in lactation, it also exerts modulatory effects on the hypothalamic-pituitary-ovarian axis. Hyperprolactinemia, even within the upper normal range, can disrupt gonadotropin-releasing hormone (GnRH) pulsatility, leading to impaired follicular development and luteal phase defects (30). In the context of PCOS, the relationship with PRL is complex and not fully understood, with some studies reporting normal or slightly elevated PRL levels in a subset of patients (31). Our results suggest that elevated PRL might be an additional, often overlooked, endocrine factor contributing to poorer IVF-ET outcomes in PCOS patients. It could potentially affect oocyte quality or endometrial receptivity. Further research is warranted to explore the mechanistic link between PRL, the metabolic and endocrine disturbances of PCOS, and implantation failure. This finding highlights the importance of a comprehensive pre-treatment endocrine evaluation, including PRL assessment, in PCOS patients planning for IVF-ET.

Although age, FSH levels, and the number of oocytes retrieved were not included in the final multivariate model, their P values were close to statistical significance, suggesting their potential influence on pregnancy outcomes should not be overlooked. Age is a well-recognized key factor affecting assisted reproduction prognosis and has been extensively demonstrated to be closely related to ovarian reserve, oocyte quality, and embryo development. Elevated FSH levels reflect diminished ovarian reserve. The number of oocytes retrieved directly indicates ovarian responsiveness during controlled ovarian stimulation and influences embryo quantity and quality to some extent. Although statistical significance was not fully reached in this study, expanding the sample size or further stratified analysis may clarify their precise roles.

This study has several limitations that should be acknowledged. First, its retrospective and single-center design may introduce selection bias and limit the generalizability of the findings. Second, although the number of clinical pregnancy events was sufficient to support the final exploratory logistic regression model according to the conventional events-per-variable principle, the overall sample size remained relatively limited. Therefore, the predictive value of irisin and other endocrine-metabolic indicators should be validated in larger multi-center prospective cohorts. In addition, BMI values were concentrated around the upper-normal/overweight threshold, and there were insufficient patients with obesity (BMI ≥28 kg/m²) to support reliable BMI-stratified or nonlinear analyses. Therefore, the absence of a significant intergroup difference in BMI should not be interpreted as evidence that BMI or obesity has no influence on IVF-ET outcomes. Third, although the analysis adjusted for several available clinical and treatment-related variables, some key determinants of IVF-ET outcomes could not be fully incorporated because of incomplete retrospective documentation. These included endometrial thickness around trigger or embryo transfer, detailed embryo quality grades, and embryo implantation scores. Although the number of transferred embryos was relatively standardized by the routine clinical protocol, the lack of detailed endometrial and embryological variables may have introduced residual confounding. For example, favorable endometrial receptivity or higher embryo quality could partly explain successful pregnancy independent of endocrine and metabolic status. Therefore, the present findings should be interpreted as associations based on available retrospective data rather than as evidence of a complete predictive model. Finally, the precise molecular mechanisms by which irisin is associated with reproductive outcomes remain to be elucidated through prospective and mechanistic studies.

Despite these limitations, our findings point to clear future clinical directions. The finding that irisin remained associated with pregnancy outcome in the multivariate model suggests its potential as a candidate biomarker for risk stratification in PCOS patients undergoing IVF-ET. Future prospective studies should aim to establish standardized reference ranges for serum irisin in this population and clarify whether changes in irisin reflect metabolic compensation, disease severity, or modifiable reproductive risk. Interventional studies are also needed before determining whether lifestyle or metabolic interventions that alter irisin levels can improve IVF outcomes. Moreover, developing a comprehensive predictive model that integrates irisin with traditional clinical, endocrine, and metabolic parameters could enhance the precision of pre-treatment counseling and treatment personalization.

## 5 Conclusion

In summary, based on available retrospective clinical, endocrine, metabolic, and treatment-related variables, this study identified irisin, LH/FSH ratio, FTI, and Gn dose as a set of predictive factors associated with clinical pregnancy outcomes in PCOS patients undergoing IVF-ET. Insulin resistance and dyslipidemia in PCOS involve complex mechanisms that may affect oocyte quality and embryo implantation through multiple pathways. These findings support the clinical relevance of comprehensive endocrine and metabolic assessment before IVF-ET and provide a basis for further development of individualized risk prediction in PCOS patients, while the independent contribution of BMI or obesity should be further evaluated in cohorts with broader BMI distributions. Future prospective studies should incorporate detailed endometrial and embryological parameters to further validate these findings and clarify how metabolic status interacts with embryo quality and endometrial receptivity in determining IVF-ET outcomes.

## Data Availability

The original contributions presented in the study are included in the article/supplementary material. Further inquiries can be directed to the corresponding author.
